# Urban safety and the role of hydrant structure and configuration

**DOI:** 10.1038/s41598-026-45799-1

**Published:** 2026-04-11

**Authors:** Vanessa Heffernan, Alan T. Murray, Kathy Baylis, Richard L. Church

**Affiliations:** 1 Department of Geography, University of California, Santa Barbara, Santa Barbara, USA; 2 Center for Spatial Science, University of California, Santa Barbara, Santa Barbara, USA

**Keywords:** Urban fires, Fire hydrants, Bivariate point pattern, Hazards, Emergency response, Fire safety, Climate sciences, Environmental social sciences, Natural hazards

## Abstract

Fire hydrants are critical to urban fire response, giving emergency personnel access to water to protect communities and property. In coastal California’s Mediterranean climate, with hot, dry summers and occasional strong winds, understanding how hydrant networks, urban form, and response operations influence structure fire outcomes is essential. This study examines past structure fire incidents in urban areas of Santa Barbara County to (i) better understand where fires occur and (ii) identify drivers of loss and occurrence, with particular attention to how hydrant infrastructure relates to these outcomes. Spatial mapping is combined with statistical models of incident counts and monetary losses. Fires are primarily clustered around major activity centers. While hydrant availability is not associated with incident occurrence, population and housing density are primary predictors of where incidents occur. Loss severity increases with total suppression time and property area, and incidents beginning near dawn have substantially higher expected losses. After controlling for operational factors and property type, hydrant proximity and code-compliant access are not independently associated with loss magnitude, suggesting that damage in this setting is more sensitive to time to control than to differences in hydrant proximity. These findings support density-focused prevention efforts and call attention to reducing suppression time to limit damage.

**Supplementary Information:**

The online version contains supplementary material available at 10.1038/s41598-026-45799-1.

## Introduction

Fire hazards are prevalent in urban environments and pose significant threats to communities worldwide. Structure fires in particular cause substantial loss of life, injuries, and property damage. The U.S. Fire Administration^1^ indicates that there were 344,600 residential building fires in the United States in 2023, resulting in 2,890 deaths, 10,400 injuries, and nearly US $11.27 billion in losses. Water is the primary extinguishing agent, and access to water is essential for limiting fire loss and spread based on lessons learned from the Great Fire of London in 1666, where thousands of buildings were lost^2^, the Great Chicago Fire in 1871, and more recently the Lahaina fire in Hawaii in 2023 and the Los Angeles fires in January 2025. During the Great Chicago Fire, the Chicago pumping stations were compromised, resulting in days of destruction across some three square miles that left nearly 100,000 residents homeless^3^. Chicago Architecture Center reports that widespread water pressure failure resulted in an estimated 300 deaths and 18,000 structures lost^4^. Early large-scale fires, such as those in London and Chicago, led to the development of water mains and the standardization of hydrant networks that exist today. Guidelines exist at many geographic scales, including international (e.g., International Code Council^5^, national (e.g., NFPA^6^, state (e.g., California Fire Code^7^, and municipal (e.g., Carpinteria-Summerland Fire Protection District^8^. Yet, despite standardized guidelines for incident response, structure fires persist and conflagrations continue to occur.

In the past, inadequate infrastructure facilitated the rapid spread of fires, resulting in significant losses. However, with the advancement of firefighting infrastructure and response measures, one might assume that such devastation would not be observed today. Unfortunately, large-scale losses by fire continue, often in and around urban areas where spread from the wildland is assisted by favorable environmental conditions, such as strong winds, dry vegetation, flammable building materials, and the close spatial arrangement of structures. While it is commonly believed that fire spread is less prevalent in densely developed environments, this does not mean that urban areas are immune. There have been several documented cases of wildfires devastating communities in urban regions. One example is the Lahaina fire in 2023, where a wildfire jumped to properties and spread relentlessly throughout the town, stopping only when it met the ocean^9^. Similarly, the Coffey Park neighborhood in Santa Rosa, California, was destroyed in 2017 by the Tubbs Fire^10^. More recently, the Palisades and Eaton fires in Los Angeles County burned for 24 days, and devastated the communities of Altadena, Pasadena, Pacific Palisades, Topanga, and Malibu, claiming 30 lives, injuring 13 people, destroying 16,251 structures, damaging 2,047 structures, and burning 37,469 acres^11^. Although fire events in densely populated urban environments may be easier to contain due to better water access, less flammable building materials, and less dry, dense vegetation, significant destruction still occurs^10^. The above events highlight that significant vulnerabilities remain, and under the right conditions, massive fire spread is possible. This means communities need to be well prepared, understand where and when incidents concentrate, which conditions are associated with larger losses, and how defensive infrastructure relates to those outcomes.

The County of Santa Barbara, known for its scenic beauty and decorated landscape, situated between the Santa Ynez foothills and the Pacific coast, faces significant wildfire vulnerabilities due to the combination of conditions that favor fire ignition and spread. Its Mediterranean climate, Sundowner winds, flammable vegetation, and other factors that contribute to wildfires make this area particularly vulnerable^12,13^. With wildfire events over the last couple of decades demonstrating greater intensity and severity, and the growing population in wildland-urban interface (WUI) communities, not only are people at risk of exposure and potential spread to urban environments, but also, human activity increases this risk. In the last century, about 95% of fires in California were human-caused^14^. Combined with a growing WUI population in California, 41% between 1990 and 2010^15^, inhabited areas are exposed to increasing levels of wildfire risk. It is imperative to conduct a thorough examination of the behavior of fires that occur in urban environments to assess defensive measures that may explain factors contributing to the structural losses experienced in fire-prone regions.

Prior work on incident patterns and structure loss spans two largely separate threads. One is urban fire-incident studies where researchers have: (i) mapped risk and hotspots, often with kernel density and related spatial tools^16–19^; (ii) linked incident counts to socio-demographic context via regression^17,20^; and (iii) modeled spatio-temporal dependence with point-process or Bayesian approaches^4,16,20–22^. The other is WUI structure-loss studies, where analyses emphasize parcel/structure and landscape factors, including defensible space, materials, topography, and housing arrangement^10,23–26^. Nonetheless, fire protection capacity has been typically represented through broader conceptual factors or proxy measures, such as proximity to fire stations, but has been less studied in terms of the spatial configuration of defensive infrastructure, specifically directly accounting for hydrant networks. Moreover, although fire incidents have been studied in different contexts, they have been tailored to specific objectives, mostly focusing on characterizing fire risk hotspots, identifying possible causes of higher incidence, predicting fire spread, or understanding the contributing factors to structural damage in the WUI. Despite advances in spatial statistics and analytical tools, the literature still lacks a more comprehensive analysis that moves beyond the descriptive characterization of fire incidence to formal tests of clustering, identifies factors associated with spatial concentration, and examines the determinants of losses while directly accounting for defensive infrastructure such as hydrant networks. This paper addresses that gap by explicitly examining hydrant infrastructure as a component of urban fire protection and by evaluating its relationship with fire incidence and structure damage in an urban region. Although this study is not designed to produce a fire-risk measure, it focuses on two empirically observable dimensions: the spatial concentration of urban structure-fire incidents and the severity of losses once incidents occur.

Hydrant code guidelines have been studied previously, delving into compliance with respect to structure access^27^ and spacing^28^. These evaluations identified vulnerabilities and revealed differential levels of code compliance, namely that commercial areas have higher compliance than certain residential neighborhoods. With spacing and structure access information combined with past fire incidents, relevant information regarding urban fire spread, patterns, damage, and response can support analysis and provide insights on response alternatives and mitigation strategies for future events. Likewise, assessing access to hydrant infrastructure is important for limiting fire spread, particularly in at-risk environments. Finally, identifying factors that make some areas more susceptible to fire and estimating expected losses can provide valuable guidance for urban planners and policymakers.

This paper introduces a framework for examining historical structure-fire incidents by examining their spatial distribution and their association with hydrant infrastructure. The analysis assesses the effectiveness of defensive actions while also characterizing patterns associated with fire-spread potential. Additionally, insights regarding the patterns of fires are sought relative to how they behave in space and how structures are destroyed based on response. The next section presents a review of the scope of this study. Then, methods and framework to analyze past incidents and their relationship with defensive infrastructure are presented. Results are detailed in the assessment of historical fires in Santa Barbara County. Finally, a discussion and concluding remarks are offered.

## Background

Most urban fire studies start with description and mapping. Official incident records are often analyzed to answer *where* and *when* events concentrate by mapping spatial hotspots and documenting temporal regularities in fire occurrence. Kernel density estimation (KDE) has been used to identify hotspots and characterize them as risk surfaces^16,17,19,21,29–35^. In several studies, these KDE-based hotspot surfaces have been paired with additional analytical methods to develop a more comprehensive understanding of urban fire patterns. For example, KDE surfaces have been combined with count models to relate spatial patterns of fire incidence to socioeconomic context^17,20^, with spatial econometric approaches to identify the socioeconomic factors associated with fire occurrence^31^, and with inferential spatial statistics, such as Diggle’s D-function and the spatio-temporal K-function to detect clustering across space and time^22^. Other studies have paired KDE mapping with average nearest-neighbor methods to characterize spatio-temporal fire patterns^18,36^, and with Getis-Ord Gi* and interpolation methods^29^.

Studies examining incident outcomes have focused on factors contributing to fire casualties through logistic regression^37^. To complement these views, ecological Poisson risk models with exposure offsets and Bayesian approaches reveal that deaths and injuries arise from disjoint populations^32,38^. Separately, research on spatial coupling of fire incidents with surrounding infrastructure and other spatial features has combined Cross-K and Cross-L functions with global and local colocation quotients to examine relationships between fire types and adjacent facilities^39^.

Beyond KDE-based hotspot mapping, K- and L-function analysis, count models, and spatial coupling, researchers have used a range of approaches to characterize urban fire patterns and relate them to neighborhood, temporal, and operational conditions. Composite-indicator methods, such as AHP-based indices, and machine-learning approaches, including supervised self-organizing maps, have been used to generate explicit fire risk assessments^40,41^. Other studies have used quad-plots and Dirichlet-multinomial models to examine how temperature, socioeconomic conditions, and temporal patterns interact with different incident types^42^. Additional spatio-temporal studies have taken a descriptive perspective focusing on spatial dependence, correlations, and clustering, and have shown that intentional or suspicious fires are associated with living condition indices and economic indicators^43,44^. Related work has also examined operational outcomes, with quantile models showing slower seasonal response times (e.g., winter) in areas with lower socioeconomic status and more complex street networks^45^. More broadly, studies associating fire incidents with neighborhood characteristics have progressed from basic regression to spatial econometric and geographically weighted regression approaches, allowing effects to vary across the urban fabric^46^, and to panel models that separate cross-sectional structure from temporal change at city or tract scale^47–49^.

Another strand of the urban fire literature emphasizes fire forecasting, operational deployment, and emergency response. Bayesian and stochastic models have been used to predict where fires are likely to occur using building footprints and past incidents, thereby avoiding some of the information loss associated with coarse spatial aggregation^50^. Markov-chain approaches indicate short-term temporal dependence, where risk tends to dip for roughly two months after a local fire, and the absence of a recent nearby event can increase short-term risk, creating windows for prevention across a region based on location^51^. Response times exhibit distinct spatial patterns. Recent predictive work integrates GIS-derived spatial features with machine learning to estimate the likelihood that an urban fire escalates, explicitly linking spatial context to response resource allocation^52^. Fire spread simulation in urban environments has also been a focal point, with several studies modeling fire dynamics in dense urban settings^53–55^. Related research has also addressed evacuation preparedness, including the development of open-source simulation frameworks^56^.

Across these streams, important gaps remain. Few studies have examined urban fire incidents through a framework that moves from hotspot identification to formal clustering analysis and then to the consequences of fire in terms of structural loss, while directly accounting for defensive infrastructure. In particular, the spatial configuration of water supply infrastructure, especially hydrant networks, remains underexamined in relation to both incident concentration and loss severity. Urban studies sometimes proxy response with station proximity or road accessibility, but direct analysis of incident-hydrant interaction remains limited. This study bridges the existing gap by combining an exploratory point-pattern analysis (KDE; univariate and cross-K and cross-L diagnostics) with count and loss models to separate where incidents happen from what drives losses, placing hydrant availability, spacing, and reach alongside density, land use, timing, and season. Thus, the paper connects urban fire analytics to the defensive infrastructure that ultimately enables suppression and limits damage in fire-prone regions.

## Methods

The goal of this study is to examine how the availability and configuration of defensive infrastructure, specifically fire hydrant networks, influence the spatial patterns of structure-fire incidents and the magnitude of losses when fires occur. To address these objectives, the analytical framework combines exploratory spatial analysis with two complementary inferential components: (i) modeling the intensity of incident counts across areal units, and (ii) modeling fire‐related losses at the property level. Both components make use of generalized linear (mixed) models tailored to the distributional nature of the outcomes, incorporating covariates that capture demographic, infrastructural, and spatial factors.

### Study area

**Fig. 1:**
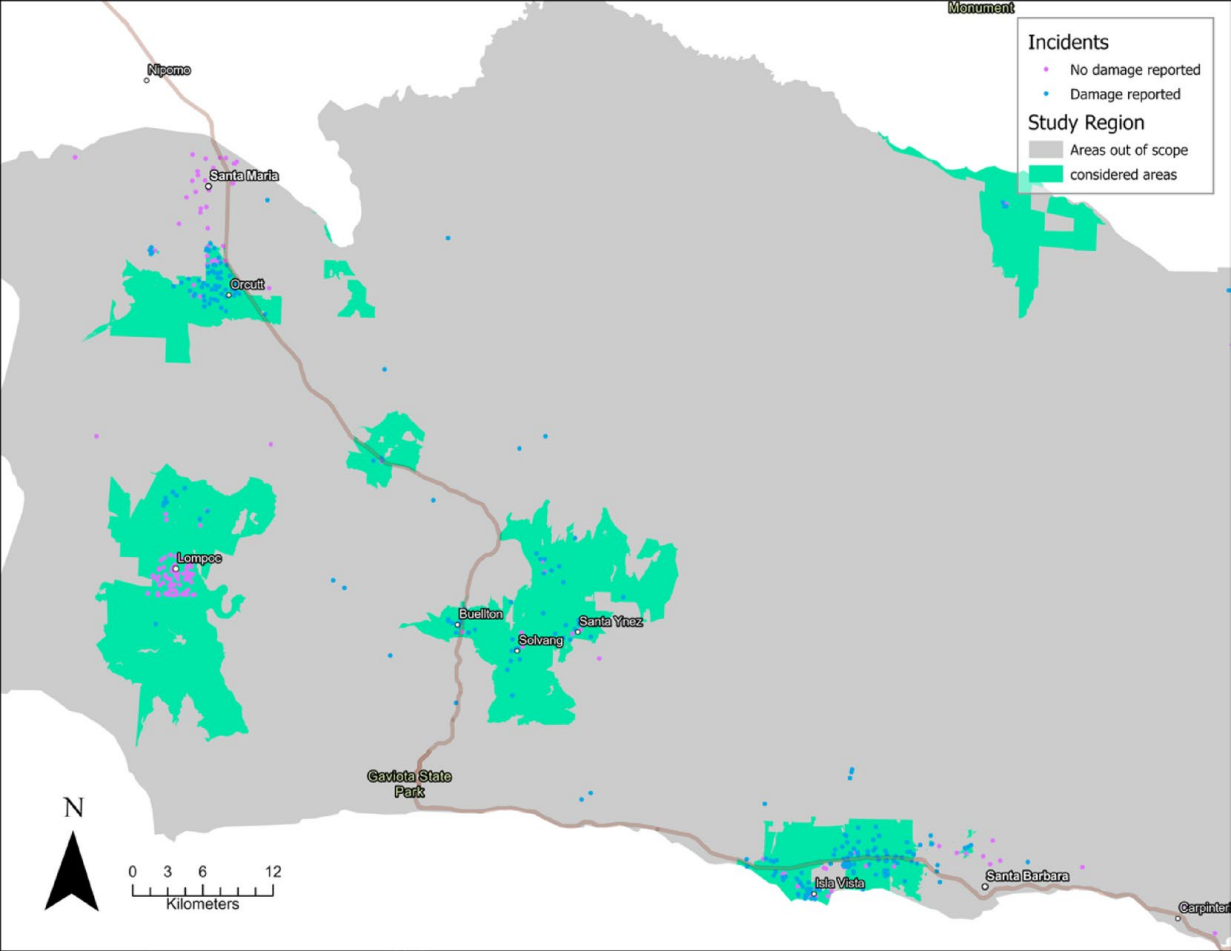
Study region in Santa Barbara County, indicating structure fires from 2014 to 2024. Map prepared in ArcGIS Pro 3.3.1 (Esri, Redlands, CA, USA; https://www.esri.com/en-us/arcgis/products/arcgis-pro/overview).

The study region encompasses urban areas within Santa Barbara County, as shown in Fig. 1. The area is aligned with the Santa Barbara County Fire Protection District’s operational scope. Incidents that occurred within jurisdictions with their own fire departments, which provide primary protection and incident reporting, were excluded since records for those jurisdictions were not available in a form that allowed a complete sample for those areas.

The urban areas in Santa Barbara County are diverse, ranging from small rural communities, such as Los Alamos and New Cuyama, to larger urban centers, such as Orcutt, Lompoc, Solvang, Goleta, and Isla Vista. For interpretation and mapping, several adjacent towns are aggregated into regional groupings. This is the case for Goleta and Isla Vista, Lompoc (including Lompoc, Vandenberg Village, Mission Hills), and Solvang (including Solvang, Buellton, Ballard, Los Olivos, Santa Ynez). These subregions differ in population size, socioeconomic composition, and settlement form, and this heterogeneity relates to the spatial patterns and model results reported in Sect. 4. Population by area is shown in Table 1 (2020 decennial census counts). Based on recent American Community Survey income estimates, Goleta, Orcutt, and the Solvang region tend to have median household incomes near or above $100k, whereas Lompoc is lower (roughly between $60 and $70k). Isla Vista is a student-dominated community (median age of approximately 21 years old), with a lower median household income of around $24,000, which is typical of college towns and reflects student composition. Ethnically, Lompoc is majority Hispanic/Latino. Goleta and Orcutt are mixed with substantial Hispanic/Latino populations. Much of the Solvang region remains predominantly non-Hispanic White. These differences matter for fire risk analysis since socioeconomic conditions and household composition can shape ignition opportunities, prevention practices, and recovery capacity^17^.

### Data

The incident dataset comprises records of structure fire responses collected by the Santa Barbara County Fire Department from January 2014 to June 2024 and was available upon request. The count of incidents in each region is reported in Table 1. Each record includes the fire’s geographic location, the number of structures involved, the incident date and time, suppression duration, and the assessed value of the affected property. Table 1 shows incident counts per region within the study area. Los Alamos and New Cuyama are excluded from the analysis due to a low incident count (eight incidents), which did not support stable spatial inference for the subregion. Hydrant locations were obtained from the Santa Barbara County Fire Department, and socioeconomic indicators (population density, housing density, median income) were derived from the American Community Survey at the census block group level. For the count models, the observation units are irregular polygons defined by the census block groups. As for the loss models, the unit of analysis is the individual fire incident.

**Table 1:** Incidents and population by region. Population: 2020 decennial census.

Region	Population	Incidents
Lompoc region	55,323	90
Goleta and Isla Vista	48,190	120
Solvang region	17,747	52
Orcutt	32,034	68
Total	153,294	330

Prior to modeling, the data underwent several derivation, cleaning, and transformation steps. The distance to the closest hydrant from each incident was calculated using road-network distance, as well as the distance to the closest fire station. Hydrant spacing was derived using the previously proposed *edge approach*^28^, which also uses road network distance. Hydrant-to-structure access compliance was also based on an approach devised in previous work^27^. Variables prone to infinite values (e.g., logarithms of densities) were filtered to retain only finite observations. Reported economic losses include property losses and content losses in U.S. dollars (USD). Continuous covariates with heavy skewness were log‑transformed using either the natural logarithm or the transformation $\:\mathrm{log}\left(1+x\right)$ to preserve zero values. Specifically, the distances to the nearest hydrant (*lh*) and fire station (*lf*), the total suppression time (*lt*), and the structure area (*la*) were transformed using this approach. Categorical variables were encoded as factors, with clear baseline categories (e.g., residential for property type, afternoon for time of day, Friday for day of week, and winter for season). All analyses were conducted in R 4.3 with dplyr, tidyr, purrr, and ggplot2 packages for data processing, analysis, and visualization. All spatial layers were transformed to a common projected coordinate reference system, NAD83 / California zone 5 (EPSG:26945).

### Exploratory analysis

A distribution of points in two-dimensional space, as in the case of structure fire incidents, can be characterized and analyzed using point pattern analysis. Such analyses can describe whether incidents are clustered, randomly distributed, or dispersed relative to complete spatial randomness (CSR). CSR corresponds to a homogeneous Poisson process and serves as the null model for inferential tests. Structure fire incidents have been studied by characterizing hotspots of incidence as a way to visualize and use the returned intensity to predict future fire incidence. The way to visualize the concentration of incidents or hotspots is commonly done by estimating an intensity surface using a two-dimensional Gaussian KDE, which generalizes the univariate KDE introduced in the statistical literature^57,58^. Given incident locations $\:\{{\mathrm{x}}_{\mathrm{i}}{\}}_{\left(\mathrm{i}=1\right)}^{\mathrm{n}}$ inside a window $\:\mathrm{W}\subset\:\:{R}^{2}$, the KDE at location $\:\mathrm{x}$ is defined as:

1 $$\hat {\lambda }\left( x \right)=\frac{1}{{2{\pi}{\sigma^2}}}\mathop \sum \limits_{{i=1}}^{n} {\mathrm{exp}}\left( { - \frac{{{{\left\| {x - {x_i}} \right\|}^2}}}{{2{\sigma ^2}}}} \right){w_i}\left( x \right),x \in W$$

where $\:{\upsigma\:}$ is a bandwidth that controls the degree of smoothing and $\:{w}_{i}\left(\mathrm{x}\right)$ is an edge‑correction weight that compensates for boundary bias. This intensity surface forms the basis for hotspot maps. The choice of $\:\sigma\:$ controls smoothness. Smaller bandwidths amplify local noise, whereas larger bandwidths can obscure compact clusters^59^. In the kernel density estimations, a $\:{\upsigma\:}=0.722\:\text{ km}$ was selected based on likelihood cross-validation and corroborated by a plug-in bandwidth criterion^60,61^. Although KDE is essential for localizing hotspots, it does not provide a formal test to assess the concentration, dispersion, or randomness of the point pattern-generating process. For this reason, although there are several ways to test against CSR (e.g., quadrat count methods and nearest neighbor point pattern measures), distance-based measures, such as Ripley’s K function and its variance‑stabilized transformation, the L function, are commonly utilized to formally quantify departures from CSR across multiple spatial scales^62–64^. The K function at radius $\:\mathrm{r}$ is defined as the expected number of additional events within distance $\:r$ of a typical event divided by the overall intensity. An unbiased estimator with edge correction is:

2 $$\:\widehat{K}\left(r\right)=\:\:\frac{\left|W\right|}{n\left(n-1\right)}\:{\sum\:}_{i=1}^{n}{\sum\:}_{j\ne\:i}\frac{I\left({d}_{ij}\le\:r\right)}{{e}_{ij}},$$

where $\:\mathrm{I}(\cdot)$ is the indicator function, $\:{d}_{ij}$ is the distance between events $\:\mathrm{i}$ and $\:j$, $\:{e}_{ij}$ is an edge‑correction factor, and $\:\left|\mathrm{W}\right|$ is the area of the observation window. Because the variance of $\:\widehat{K}\left(\mathrm{r}\right)$ increases with $\:r$, the L function was proposed^64^:

3 $$\:\widehat{L}\left(r\right)=\sqrt{\frac{\widehat{K}\left(r\right)}{{\uppi\:}}},$$

which stabilizes the estimator’s variance. Under CSR, the theoretical value of $\:\mathrm{L}\left(\mathrm{r}\right)$ is $\:r$. It is therefore common to examine the centered function $\:\widehat{L}\left(r\right)-\mathrm{r}$. Positive values indicate clustering, whereas negative values indicate dispersion. Monte Carlo simulation envelopes are used based on CSR to assess the significance of observed deviations^65^. In addition to analyzing incident clustering alone (univariate case), the bivariate cross-L function is used to assess bivariate patterns (the relationship between two point patterns) by examining the spatial relationship between incident and hydrant locations. The cross-K function $\:{K}_{IH}\left(\mathrm{r}\right)$ measures the expected number of hydrants within a distance $\:\mathrm{r}$ of a typical incident, standardized by the mean density of hydrants. The cross-L function is defined analogously as $\:{L}_{IH}\left(r\right)=\sqrt{{K}_{IH}\left(r\right)}/{\uppi\:}$. Comparing $\:{\widehat{L}}_{IH}\left(\mathrm{r}\right)$ with its CSR expectation $\:r$ allows for testing whether hydrants are randomly located relative to incidents or whether there is evidence of attraction (clustering) or repulsion (dispersion). Throughout, cross functions are computed using Euclidean distance. An important consideration for analysis and interpretation concerns the assumption of the null model, since it implies a homogeneous Poisson process. In the present context, CSR should not be interpreted as a literal model of urban structure. Urban fire incidents arise in a built environment shaped by land use, road geometry, and uneven settlement patterns. Rather, CSR should be used as a benchmark to test whether the observed point pattern is more clustered or more dispersed than expected under spatial homogeneity. Departures from CSR, therefore, indicate non-random spatial structure, but do not by themselves identify the mechanism producing that structure.

### Modeling incident counts

Fire incidence in urban areas is associated with socioeconomic factors, including low income, high unemployment rates, household overcrowding, and ethnicity, where higher incidence and casualties are more prevalent in economically deprived areas^17^. Given the importance of socioeconomic variables in fire incidence, Poisson regression provides a way to assess variables related to spatial clustering or perceived spatial distribution of incidents. A Poisson model is suitable because the response variable is the number of incidents observed in each census block group. Thus, a count regression provides the natural starting point. Let $\:{Y}_{i}$ denote the number of structure fire incidents observed in polygon $\:i$ during the study period. A Poisson regression is defined as:

4 $$\log \left( {\lambda _{i} } \right) = \beta _{0} + x_{i}^{{ \top }} \beta + \log \left( {a_{i} } \right),$$

where $\:{Y}_{i}|{x}_{i}\sim\:\mathrm{Poisson}\left({\lambda\:}_{i}\right)$, $\:{\lambda\:}_{i\:}=\:\mathbb{E}\left({Y}_{i\:}\right|{x}_{i\:})$, $\:{x}_{i}$ is a vector of covariates, $\:{a}_{i}$ is the polygon area in square feet used as an offset, and $\:\beta\:$ are parameters to be estimated. However, inspection of the Poisson model residuals reveals that substantial overdispersion exists when the Pearson dispersion $\:\varphi\:$ > 1, indicating that the variance greatly exceeds the mean. Conventional guidance suggests the adoption of negative binomial (NB) models to resolve this issue:

5 $$\mu _{i} = \exp \left( {\beta _{0} + x_{i}^{{ \top }} \beta } \right)a_{i} ,$$

where $\:{Y}_{i}\sim\:N\mathrm{B}\left({{\upmu\:}}_{i},\:{\upkappa\:}\right)$. Count models are estimated in R using the glm() function for the Poisson specification, and the glm.nb() function from the MASS package for the negative binomial specification.

### Modeling fire-related losses

The second modeling task seeks to explain variation in monetary losses and the importance of the hydrant infrastructure, measured by distance to the incident and by hydrant-to-structure access compliance, conditioned on incident. Let $\:{L}_{j}$ denote the total economic loss for incident $\:j$ in U.S. dollars. Because fire-related losses are positive, continuous, and strongly right-skewed, a Gaussian model would be inappropriate. Accordingly, losses are modeled using a Gamma specification with a log link, and a block-group random intercept is included to account for clustering and unobserved area-level heterogeneity. Specifically:

6 $$L_{j} {\mid }x_{j} ,b_{{g\left( j \right)~}} \sim \mathrm{Gamma}\left( {\mu _{j} ,\phi } \right),\,\mu _{j} = {\mathbb{E}}\left[ {L_{j}{\mid } x_{j} ,b_{{g\left( j \right)~}} } \right],\,\log \left( {\mu _{j} } \right) = \beta _{0} + x_{j}^{{ \top }} \beta + b_{{g\left( j \right)~}}$$

where $\:g\left(j\right)$ indexes the census block group corresponding to incident $\:j$. A random intercept $\:{b}_{g\left(j\right)}\sim\:N(0,{\sigma\:}^{2})$ captures unobserved heterogeneity across block groups, reflecting that nearby properties may share latent risk factors (e.g., building codes or vegetation). Here, $\:{\mu\:}_{j}$ denotes the conditional mean loss for incident $\:j$, $\:\phi\:$ is the Gamma dispersion parameter, and $\:{x}_{j}$ is the vector of covariates. The log link ensures that multiplicative effects of covariates on expected loss translate into additive effects on $\:\mathrm{l}\mathrm{o}\mathrm{g}\left({\mu\:}_{j}\right)$. Covariates in $\:{x}_{j}$ include the log of the distance from incident $\:j$ to its closest hydrant, the log of the distance from incident $\:j$ to its closest fire station, the log of total suppression time in minutes, and the log of the area of the property where the incident occurred. Some categorical variables are included, such as hydrant-to-building code compliance (1 if structure where the incident occurred was compliant and 0 otherwise), building type (residential, commercial, outdoors building, etc.), spatiotemporal variables, such as time of the day in which the incident occurred (dawn, morning, lunch time, afternoon, evening, and night), day of the week (Monday to Sunday), and season (winter, spring, summer, and fall). Loss models are estimated in R using glmmTMB() from the glmmTMB package, specifying a Gamma distribution with a log link.

## Results

**Fig. 2:**
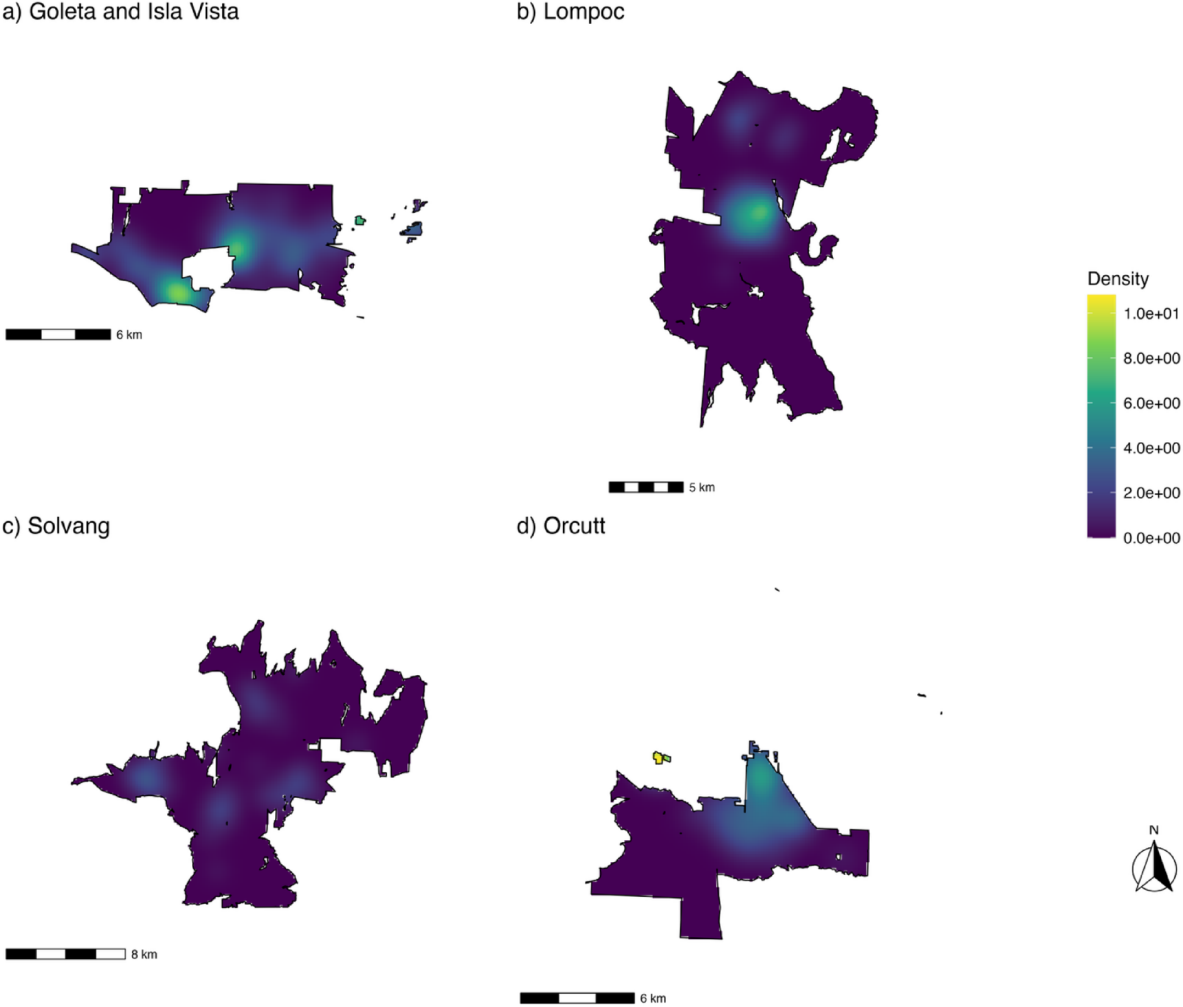
Kernel density for the areas by subregion. (Bandwidth σ = 0.722 km; incidents per km^2^).

This section reports the findings in three stages aligned with the research aims. First, an exploratory point pattern analysis shows incident clusters and spatial scale. Second, count models show the relationship between hydrant presence and fire intensity, as well as which factors are associated with incident intensity. Lastly, loss models identify factors associated with economic loss during an incident, including hydrant infrastructure. The estimates, shown in Fig. 2, reveal incident concentrations, or hotspots, within the study region, with these hotspots primarily around the principal activity centers. For example, the area of Isla Vista and Goleta presents a couple of compact core hotspots with density $\:\lambda\:\left(x\right)$ peaking near 9 incidents per square kilometer in Isla Vista and downtown Goleta, while incidents decline to less than 1 per square kilometer towards the peripheries of those hotspots. Similarly, in the Lompoc region, the incidence is concentrated in downtown Lompoc, with density $\:\lambda\:\left(x\right)$ peaking at nearly 7 incidents per square kilometer. In the Solvang region, the highest density peaked in Buellton at 3 incidents per square kilometer, where the hotspots were present in downtown Buellton (see Fig. 1 to locate these towns within the study region), and with less density in downtown Solvang, downtown Santa Ynez, and Los Olivos. The highest density was found in the Orcutt region, within Tanglewood, with an intensity of nearly 11 incidents per square kilometer, followed by a hotspot in the northern part of Orcutt.

**Fig. 3:**
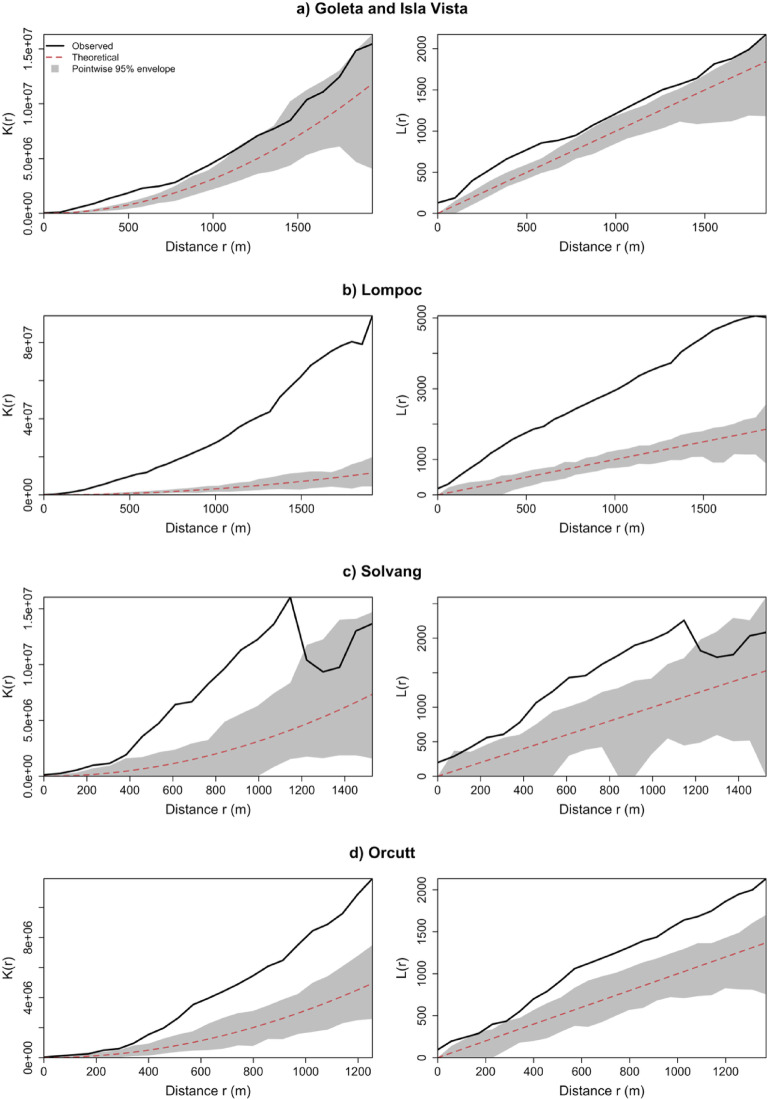
K and L functions for the subregions within the study area.

Since KDE is merely descriptive, departures from CSR were formally evaluated within each subregion using Ripley’s K and L functions with simulation envelopes. Figure 3 shows that clustering within each subregion is statistically significant across a range of distances. Across the four subregions, the observed $\:L\left(r\right)$ consistently departed from the theoretical CSR expectation, confirming the nonrandomness of the incident’s spatial distribution. These subregions exhibited clustering at characteristic spatial scales, with the magnitude and spatial extent of clustering varying with settlement size and morphology. Particularly, in the subregion of Isla Vista and Goleta, the $\:L\left(r\right)$ curve lay substantially above the CSR envelope across nearly the full distance range shown, indicating persistent clustering from local to broader neighborhood scales. This is consistent with the hotspots revealed by the kernel density analysis. Likewise, in the Lompoc region, the $\:L\left(r\right)$ curve exceeds the envelopes up to 2 km. This indicates statistically significant clustering across local and neighborhood scales. This scale-wide departure is consistent with multiple local hotspots superimposed on a broader urban concentration. In the Solvang region, the observed $\:L\left(r\right)$ exceeds the CSR band up to 1.2 km, indicating statistically significant clustering at short- to medium-range scales, consistent with the multiple centers contained in this region. In Orcutt, the clustering is statistically significant up to 1.5 km. This shows the concentration at the local scale and neighborhood scale.

**Fig. 4:**
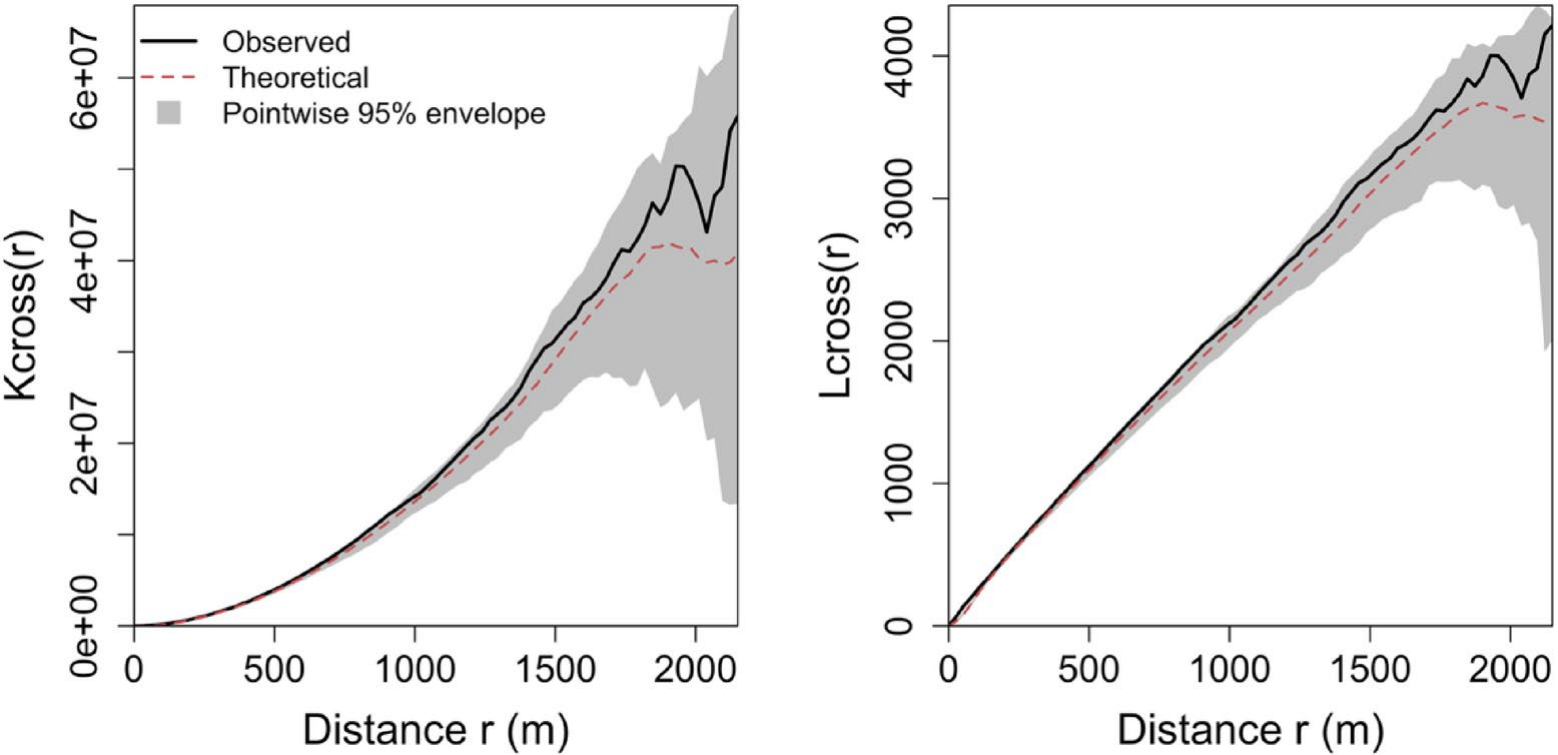
Cross-K and L functions for the study region.

Now that the clustering of the incidents is statistically confirmed, the subsequent analyses examine whether hydrant locations are spatially associated with incidents and whether hydrant-related, demographic, and operational factors help explain incident intensity and loss severity. Hydrants, as the first line of defense, are a mitigation tool rather than a preventive method. Figure 4 displays the cross-K and L functions, which consider a bivariate pattern between incident and hydrant locations to statistically test whether the locations of hydrants and incidents exhibit clustering or dispersion across space, or whether their locations are uncorrelated.

The cross $\:K\left(r\right)$ and $\:L\left(r\right)$ functions remain within the simulation envelope across the distance range considered, indicating no statistical evidence of departure from the baseline null model of spatial independence between incident and hydrant locations. In other words, the observed incident and hydrant point patterns do not exhibit detectable attraction or repulsion in the baseline planar analysis. This is consistent with the count models shown in Table 2, where hydrant covariates were not statistically significant.

**Table 2:** Negative Binomial (NB) regression models for incident intensity.

Term	Model 1 (NB)	Model 2 (NB)
Intercept	-16.963*** (1.771)	-17.725*** (1.510)
Hydrant count	0.002 (0.004)	0.004 (0.004)
log(Income)	-0.183 (0.144)	-0.196 (0.144)
log(House density)	0.834*** (0.048)	—
log(Pop. density)	—	0.833*** (0.047)
AIC	569.3	565.7
BIC	584.3	580.7
logLik	-279.6	-277.9
Dispersion (Pearson $\:\varphi$)	1.307	1.245
DHARMa KS (D, p)	0.048, 0.891	0.091, 0.170
DHARMa Disp (stat, p)	0.929, 0.784	0.946, 0.896
DHARMa Zero inf. (stat, p)	0.934, 0.768	0.917, 0.664
DHARMa Outlier (stat, p)	0.000, 0.940	1.000, 1.000
Pseudo-*R*^2^ (McFadden)	0.188	0.193
Pseudo-*R*^2^ (Nagelkerke)	0.588	0.598
Residual Moran’s I (stat, p)	0.073, 0.066	0.135, 0.004

Standard errors in parentheses. Significance *p* < 0.05 *, *p* < 0.01 **, *p* < 0.001 ***.

Following the spatial point pattern analysis, regression models were estimated to assess whether hydrant infrastructure, income, and density measures (house and population) systematically explained the observed variation in incident counts at the census block group level. A Poisson GLM was first fitted but exhibited severe overdispersion (Pearson $\varphi=3.29$), confirming that it was an inadequate baseline model. For clarity and parsimony, results from the Poisson GLM are not displayed, as subsequent models provide more reliable inference.

A suite of models was estimated with different combinations of covariates to probe the drivers of incident counts, and the two most relevant are shown in Table 2. Explanatory variables included hydrant count within each census block group (Hydrant count), population density (Pop. Density), housing density (House density), and median household income (Income). All density measures were expressed in logarithms to linearize elasticities, and a logarithmic area offset ensured that intensity is modeled, i.e., incidents per unit area. Model selection relied on AIC/BIC and DHARMa residual diagnostics (uniformity and dispersion). Across specifications, density is the only strong predictor of incident intensity, i.e., house density in model 1 and population density in model 2. The two density measures are highly correlated at the census block-group level (Pearson $\:r\:=\:0.973$), indicating that they capture closely related aspects of spatial concentration. Whether operationalized as housing density or population density, the density coefficient remains positive, similar in magnitude, and highly stable across the two specifications. For example, in the population density model, the coefficient is $\:\beta\:=0.833$ statistically significant, suggesting that a 1% increase in population density is associated with a 0.83% increase in expected incident intensity, holding other factors constant. By contrast, hydrant count and income were not statistically significant in either specification. This suggests that hydrant availability, as represented here by hydrant count at the census block-group level, does not explain incident intensity once density and income are taken into account. Similarly, income does not explain where incidents occur at this spatial scale (see Sect. 5), even though it may affect the economic magnitude of losses. DHARMa diagnostics indicate well‑behaved residuals and no evidence of residual overdispersion in either model. Residual spatial autocorrelation was evaluated using Moran’s I with queen-contiguity weights at the census block-group level. Model 1 showed weak residual spatial autocorrelation, while Model 2 retained modest positive residual spatial autocorrelation. A spatial negative binomial model was estimated as a robustness check, but it was not reported because the coefficient values and significance did not depart significantly from the models reported in Table 2.

Since the presence of hydrants did not seem to influence the location of incidents or their intensity, it is crucial to examine how hydrants contribute to fire damage, i.e., once an incident occurs. Table 3 presents the estimates of the Gamma models with a log link, estimated as a sequence of specifications that progressively add covariates while retaining a random intercept for census block group (GEOID) to capture unobserved location-level heterogeneity. The loss-model sample includes 90 incidents $\:(n=90)$ for which complete positive-loss information was available. Because this complete-case loss sample is substantially smaller than the full incident dataset, the loss-model results should be interpreted cautiously. Coefficients are presented on a log scale, and all models include a random intercept. AIC and BIC were computed for each specification, along with the log‑likelihood. Marginal and conditional $\:{R}^{2}$ values were obtained using the approach of Nakagawa and Schielzeth. The marginal $\:{R}^{2}$ reflects the variance explained by fixed effects alone, while the conditional $\:{R}^{2}$ accounts for fixed and random effects. The intraclass correlation coefficient (ICC) measures the proportion of total variance attributable to the random intercept. DHARMa residual diagnostics for uniformity, dispersion, and outliers, as well as quantile tests, were conducted to assess model adequacy. Models with lower AIC/BIC, higher $\:{R}^{2}$, and acceptable DHARMa diagnostics were considered to provide a better overall fit. Model 3 provides the best overall balance of fit and parsimony. However, because the fully adjusted specification includes the largest number of parameters relative to the available loss sample, bootstrap-based stability checks were applied to Model 5 to assess the robustness of its coefficients under finite-sample conditions.

**Table 3:** Gamma regressions with a log link.

$\:Y=\:\mathrm{L}\mathrm{o}\mathrm{s}\mathrm{s}\:\left(\mathrm{U}\mathrm{S}\mathrm{D}\right);\:\mathrm{G}\mathrm{a}\mathrm{m}\mathrm{m}\mathrm{a}\left(\mathrm{l}\mathrm{o}\mathrm{g}\right);\:n=90$	Model 1	Model 2	Model 3	Model 4	Model 5
(Intercept)	2.956 (3.151)	4.770 (3.354)	4.098 (3.215)	4.537 (3.174)	4.724 (3.385)
*lh*	-0.038 (0.257)	-0.243 (0.273)	-0.393 (0.259)	-0.435 (0.250)	-0.495 (0.258)
*la*	0.542** (0.172)	0.408** (0.182)	0.310 (0.174)	0.260 (0.170)	0.215 (0.177)
*lf*	-0.076 (0.350)	-0.024 (0.357)	0.202 (0.320)	0.176 (0.301)	0.233 (0.314)
*lt*	1.213*** (0.185)	1.228*** (0.188)	1.220*** (0.181)	1.330*** (0.187)	1.326*** (0.189)
*Hyd-to-str* (baseline: noncompliant)	0.001 (0.411)	—	—	—	—
Type of building (baseline: residential)					
Commercial	—	0.260 (0.636)	0.350 (0.581)	0.344 (0.546)	0.223 (0.550)
Institutional	—	1.540 (1.674)	1.529 (1.584)	1.576 (1.510)	1.990 (1.545)
Outbuilding	—	-1.317* (0.669)	-1.431* (0.633)	-1.739** (0.643)	-1.799** (0.686)
Time of the day (baseline afternoon)					
Dawn	—	—	1.454* (0.607)	1.363* (0.616)	1.463* (0.651)
Morning	—	—	0.200 (0.554)	0.303 (0.577)	0.386 (0.579)
Lunch Time	—	—	0.100 (0.726)	-0.063 (0.738)	-0.061 (0.767)
Evening	—	—	-0.952 (0.625)	-1.116 (0.667)	-0.941 (0.689)
Night	—	—	0.393 (0.554)	0.426 (0.591)	0.424 (0.596)
Day of the week (baseline Friday)					
Monday	—	—	—	-0.134 (0.792)	-0.101 (0.829)
Tuesday	—	—	—	-0.153 (0.691)	0.025 (0.688)
Wednesday	—	—	—	0.023 (0.657)	0.193 (0.656)
Thursday	—	—	—	0.510 (0.670)	0.693 (0.676)
Saturday	—	—	—	1.304 (0.762)	1.634* (0.802)
Sunday	—	—	—	-0.337 (0.734)	-0.186 (0.722)
Season of the year (baseline Winter)					
Spring	—	—	—	—	-0.373 (0.502)
Summer	—	—	—	—	0.189 (0.543)
Fall	—	—	—	—	-0.421 (0.441)
AIC	2084.9	2084.29	2080.51	2083.94	2088.17
BIC	2104.9	2109.29	2118.01	2136.43	2148.17
logLik	-1034.4	-1032.15	-1025.25	-1020.97	-1020.09
*R*^2^ (marginal)	0.432	0.457	0.571	0.638	0.648
*R*^2^ (conditional)	0.774	0.768	0.796	0.781	0.787
ICC (adjusted)	0.602	0.573	0.524	0.397	0.395
DHARMa KS (D, p)	0.11, 0.26	0.07, 0.72	0.08, 0.61	0.06, 0.88	0.07, 0.80
DHARMa Disp (stat, p)	1.16, 0.27	3.36, 0.14	0.79, 0.30	1.41, 0.27	2.24, 0.17
DHARMa Out (stat, p)	1.00, 0.51	0.00, 1.00	1.00, 0.51	0.00, 1.00	1.00, 0.51
DHARMa Quant (p)	0.212	0.138	0.316	0.310	0.074

Standard errors in parentheses. Significance *p* < 0.05 *, *p* < 0.01 **, *p* < 0.001 ***. *lh*: distance to the nearest hydrant; *la*: structure area *lf*: distance to the nearest fire station; *lt*: total suppression time; *Hyd-to-str*: hydrant to structure access.

Across the five models, total suppression time emerged as the most consistent predictor of loss severity. Once time-of-day controls were introduced, incidents beginning at dawn, relative to the afternoon baseline, were also associated with substantially higher expected losses, while outbuildings were associated with significantly lower expected losses than the residential baseline. By contrast, property area was positive in the simpler specifications but became less precisely estimated in the richer models. The coefficient for the log of total suppression time is large and highly significant in every specification, ranging from 1.213 to 1.330. In the preferred specification (Model 3), the suppression-time coefficient is 1.220, implying a strong positive association between longer operational duration and higher expected loss. This implies that a 10% increase in suppression time is associated with roughly a 12% increase in expected loss, and that doubling suppression time is associated with an expected loss about 2.3 times as large. This effect remains stable across specifications, indicating that operational duration is the most consistent correlate of loss severity once an incident occurs.

**Fig. 5:**
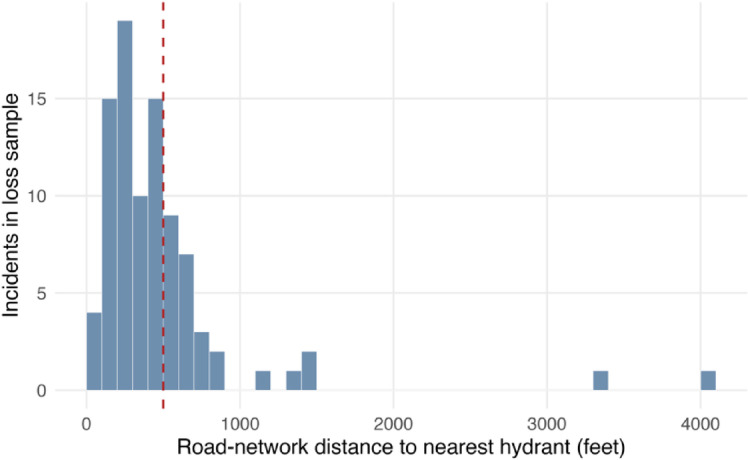
Distribution of incident-to-nearest-hydrant distance.

Other covariates show more limited or context-dependent associations. Distances to hydrants, to fire stations, and hydrant-to-structure access compliance are not statistically different from zero. This suggests that while hydrant networks are critical for fire response, proximity alone does not fully capture their role in explaining variation in loss magnitude once an incident is underway. To better interpret the null distance coefficients, Fig. 5 summarizes the distribution of nearest-hydrant distances based on road-network distance. The dashed line denotes the 500 ft (152.4 m) mark. The distribution is concentrated at relatively short distances, with a median of 363.7 ft (110.9 m), an interquartile range of 213.1-543.8 ft (65.0–165.8 m), and 70.0% of incidents occurring within 500 ft of a hydrant. In addition, 42.2% of incidents occur within 300 ft (91.4 m). This range of variability can explain the inability of proximity to directly capture hydrant efficacy. Accordingly, the null coefficient is better interpreted as limited identifiability for the measured proximity proxy than as evidence that hydrant proximity is unrelated to loss severity.

Time-of-day effects are more pronounced. Holding other included covariates constant, incidents beginning at dawn are associated with expected losses approximately 3.9 to 4.3 times those of afternoon incidents across Models 3–5. Regarding property type, commercial and institutional categories are not statistically distinguishable from the residential baseline, whereas outbuildings are associated with significantly lower expected losses. This likely reflects the smaller size and lower economic value of outbuildings relative to the residential baseline.

Given the limited positive-loss sample of 90 incidents across 54 block groups, coefficient stability in the fully adjusted Gamma mixed model (Model 5) was assessed using a 500-resample nonparametric bootstrap (Table 4). Model 5 estimates 24 parameters, corresponding to approximately 3.75 observations per parameter, which warrants caution regarding finite-sample sensitivity. All bootstrap resamples converged, although only 109 refits (21.8%) yielded a positive-definite Hessian, indicating that the most complex specification is numerically sensitive. Among the coefficients examined, suppression duration was the most stable predictor, while property area showed moderate stability. By contrast, the distance-related coefficients were materially less stable, with wide intervals and lower sign stability. These results suggest that the fully adjusted loss model should be interpreted cautiously, with the strongest support concentrated in suppression duration and, to a lesser extent, property area. Despite the robustness of the suppression-time coefficient, it should be interpreted as an association rather than a causal effect, since more severe incidents may themselves require longer suppression. The comparatively low stability of the distance-related coefficients is consistent with the limited variability in nearest-hydrant distance within the loss-model sample. Because the observed distances are concentrated within a relatively short range, the model has limited leverage to identify a stable distance effect, making those coefficients more sensitive to sample variation.

**Table 4:** Coefficient stability assessment for Model 5.

Coefficient	Estimate	95% bootstrap CI	Sign stability
Total suppression time	1.326	[0.452, 1.948]	99.10%
Property area	0.215	[-0.103, 1.441]	91.70%
Distance to closest fire station	0.233	[-1.135, 2.209]	74.30%
Distance to closest hydrant	-0.495	[-1.849, 0.412]	85.30%

Model fit and diagnostics broadly support these conclusions. Information criteria improve as key covariates are added, with Model 3 attaining the lowest AIC among the reported specifications and thus providing the best overall balance of fit and parsimony. Models 4 and 5 add further temporal controls and preserve the main substantive pattern, namely a strong association with suppression duration, a positive dawn effect, and weak distance-related terms. The random intercept for GEOID remains empirically important throughout. The ICC declines from 0.60 in Model 1 to approximately 0.40 in the richer specifications, indicating that observed predictors explain part, but not all, of the between-place variability. Likewise, marginal $\:{R}^{2}$ increases across specifications, while conditional $\:{R}^{2}$ remains higher, showing that the GEOID random intercept continues to capture meaningful place-specific heterogeneity beyond the observed covariates.

## Discussion

Several aspects require further discussion. Firstly, there are several considerations associated with modeling insights. Prevention and outreach efforts would be most effectively prioritized in dense urban areas where incident intensity is concentrated, while expectations that hydrant counts will reduce incident occurrence should be tempered. Because higher losses are strongly associated with longer suppression time, investment in measures that shorten the time needed to control a fire, such as staffing, dispatch, access, alternate water supply, pressure reliability, and operational preplans, may help reduce damage. The positive association between dawn incidents and loss severity suggests that shifts, coverage, or alerting protocols in the early morning hours warrant closer attention.

Furthermore, there is a very important aspect in the practical implications of the results. The fact that the proxies used to measure the importance of hydrant infrastructure were not directly explanatory of the losses should be interpreted carefully. This null association should not be interpreted as evidence that hydrants are unimportant. Rather, it suggests that simple spatial availability metrics (water access to structure/proximity) may not capture the mechanisms by which water-supply systems improve outcomes. In this dataset, context reveals the nuances of these results. Within the loss-model sample $\:(n\:=\:90)$, hydrant coverage is already dense. The median distance from an incident to the closest hydrant is 363.7 ft (110.9 m), and 70% of the incidents occurred within 500 ft (152.4 m) of a hydrant. With such limited variation in hydrant distance, there is little statistical leverage to detect an effect, even if hydrants matter operationally. In addition, responding apparatus typically provide an immediate initial water supply from onboard tanks (often on the order of hundreds of gallons, depending on engine type), allowing crews to begin suppression while a sustained hydrant supply is established. From the dataset, there was no evidence of fire spread between units, which is consistent with a system that generally contains incidents effectively. A more informative interpretation is that hydrant systems may influence losses through mechanisms that proximity alone cannot capture, such as whether adequate flow and pressure can be delivered when needed, how reliable the system is under demand, and the operational constraints crews face in establishing a sustained water supply. System layout and the placement of water supply infrastructure have been shown to be crucial to determine whether protection requirements can be met^66^. In that light, it is not surprising that operational time is such a strong predictor in the loss models. Response and suppression durations likely reflect (among other things) how quickly a sustained water supply is secured and the incident is brought under control. Accordingly, hydrant planning remains essential for mitigation, but its benefits in this setting may be realized primarily through faster suppression and reduced escalation.

Secondly, the modifiable areal unit problem (MAUP) is a matter of further discussion for the negative binomial regressions. The spatial scale at which the socioeconomic variables were considered was the Census group level, which revealed that at this scale, population and house density were the primary factors driving incident intensity. However, at other scales, other variables can also play a significant role, and since these variables are not present at a more aggregated spatial scale, some insights might not be identified. This is also noted and discussed in previous works^17^, where the strength of the relationships between population characteristics and fire rate variations was dependent on the unit of analysis. For this reason, additional regressions were performed in which the space was partitioned into grids of 500 m x 500 m, 725 m x 725 m, and 1 km x 1 km. Census variables were interpolated from the census block group, and the remaining variables (related to hydrant infrastructure) were aggregated at this level. The significance of population and the number of housing units remained, and income at these levels was also significant, as shown in Table 5, indicating that at these spatial scales, areas with lower income are associated with higher fire incidence.

**Table 5:** Poisson models with different grid sizes.

y: Incident rate	500 m		725 m		1000 m	
Term	Model 1	Model 2	Model 3	Model 4	Model 5	Model 6
Hydrant spacing (m)	-0.00004	-0.00004	-0.00003	-0.00003	-0.00013	-0.00013
	(-0.00006)	(-0.00006)	(-0.00006)	(-0.00005)	(-0.00008)	(-0.00009)
log(Population)	0.478***	––	0.549***	––	0.604***	––
	(-0.056)		(-0.056)		(-0.061)	
log(Housing units)	––	0.502***	––	0.581***	––	0.637***
		(-0.059)		(-0.059)		(-0.063)
log(Income)	-0.537***	-0.539***	-0.489***	-0.512***	-0.583***	-0.610***
	(-0.128)	(-0.127)	(-0.137)	(-0.134)	(-0.137)	(-0.134)
AIC	1344.9	1344.5	1034.3	1029.8	826.7	815
Dispersion (Pearson $\:\phi\:$)	0.885	0.884	0.996	0.988	1.227	1.195
Pseudo-$\:{R}^{2}$ (McFadden)	0.624	0.624	0.711	0.713	0.77	0.773
Sample size (N)	953	953	568	568	370	370

Standard errors in parentheses. Significance *p* < 0.05 *, *p* < 0.01 **, *p* < 0.001 ***.

Thirdly, it is consistent with the literature that incident intensity is associated with house and population density. Incidents are more likely to occur where residential and human activity revolve. However, other socioeconomic variables can untangle which characteristics of the population are associated with nuances of this intensity, and, in a larger sample, this can be further studied.

**Fig. 6:**
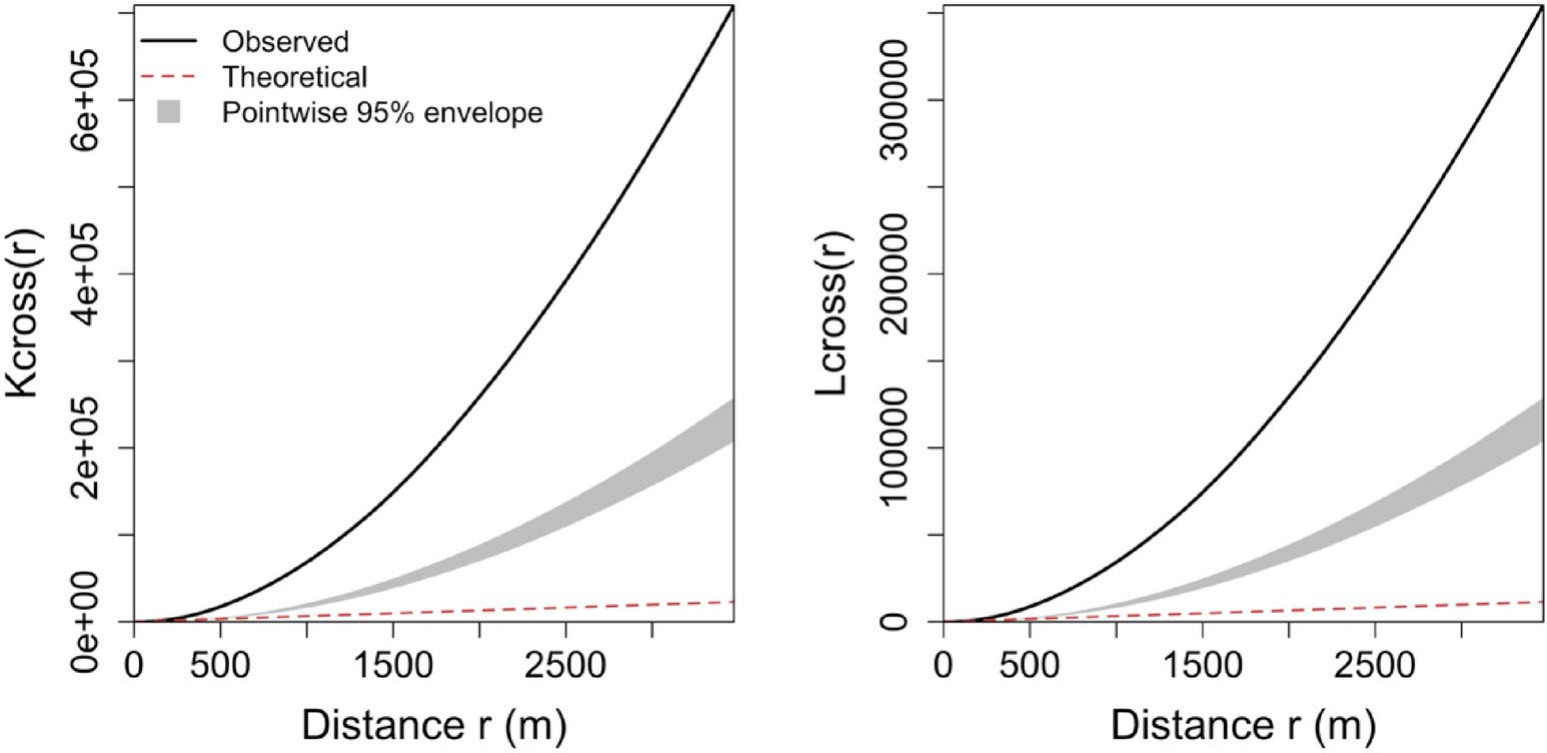
Linear-network Cross-K and L functions for the study region.

Fourthly, there are some aspects regarding point pattern analysis assumptions and diagnostics that need to be carefully noted. The bivariate cross‑functions for incidents and hydrants were computed in the plane, whereas hydrants are constrained to street networks. Computing a linear-network cross-K (assuming that both patterns are constrained to the road network) produces the curve depicted in Fig. 6, lying above the simulation envelope over most distances, indicating stronger incident-hydrant co-location than expected under a simple random-on-network null. However, this interpretation has important limitations. To compute the linear-network cross-K, incident locations must be projected onto the road network, even though fire incidents occur at structures rather than anywhere along the road centerlines. This suggests a new research avenue for assessing the co-location of patterns across different spatial domains, i.e., when one pattern can occur *anywhere* in space and another is restricted to a linear network.

Finally, several measurements introduce unavoidable uncertainty. Hydrant variables capture presence and proximity, but not flow, pressure, operability, valve size, or main diameter. These unobserved attributes likely influence performance and operational time to control a fire. Incident loss values reflect assessed or reported losses and may contain measurement error or under/over reporting. Temporal covariates (e.g., dawn) may capture detection and mobilization delays or reflect other causes of fire (fire while people were asleep, under the influence of narcotics or alcohol, etc.) rather than intrinsic fire behavior. The loss models were estimated on the subset of incidents with complete loss data, and unobserved confounding (e.g., construction materials, interior contents, wind, and weather at ignition) cannot be excluded. Also, sample sizes were limited in some subregions and for some loss categories, which constrains precision.

Notwithstanding these caveats, the main findings were consistent across analytic lenses. Incidents cluster with urban concentration, hydrant-related variables do not independently explain incident intensity at the block-group scale, and once incidents occur, higher losses are most consistently associated with longer suppression duration, with additional evidence of a dawn effect and more limited evidence for property area. These results provide an empirical basis for prioritizing density‑focused prevention and mitigation focused on operational time in fire‑prone urban regions.

## Conclusions

Although wildfires are the primary concern for the spread of fire from wildland to urban areas in at-risk regions, they can also occur in urban settings under the right environmental conditions and spread across communities due to inherent vulnerability. This study shows how spatial statistical tools can help characterize where structure fires concentrate, the drivers of concentration, and which factors are most strongly associated with losses once an incident occurs, with particular attention to how hydrant infrastructure relates to these outcomes. The results suggest that incident intensity is primarily related to the concentration of people and structures, while loss severity is most consistently associated with operational duration. Property area shows a positive association in simpler loss specifications, but that relationship is less stable once richer temporal controls are added. In addition, incidents beginning near dawn are associated with substantially higher expected losses than afternoon incidents in the richer loss specifications. These findings support prioritizing prevention and outreach in high-density areas at neighborhood-relevant scales and strengthening measures associated with time-to-control, e.g., staffing, access, dispatch efficiency, and water supply reliability, especially for larger properties where potential losses are greater. Importantly, the lack of a direct association between hydrant proximity/access measures and loss magnitude should not be interpreted as indicating that hydrants are unimportant. Instead, it is consistent with a setting where hydrant access is generally already present for most incidents, so the measured distance proxies provide limited leverage for explaining variation in loss magnitude. In that sense, the null distance effect may reflect a system that meets baseline coverage needs, while variation in losses is associated with how quickly a sustained water supply is established and the incident brought under control. Although the association between loss severity and suppression duration and exposed property scale is not unexpected, the result remains informative because it clarifies which variables carry the strongest empirical signal in the loss models after adjustment for the other covariates. Suppression duration, in particular, was the most stable predictor. However, this finding should be interpreted cautiously, as suppression duration may also reflect the incident’s underlying severity or complexity. Thus, its value is less as a causal estimate than as an indicator of the operational conditions under which larger losses are observed.

Future research could extend the present framework in three directions. First, the empirical components analyzed here, namely incident concentration and conditional loss severity, could serve as building blocks for more comprehensive urban fire-risk measures that integrate observed fire patterns, defensive infrastructure, and broader exposure and vulnerability conditions. Second, preparedness and response planning merit closer attention, including the role of evacuation training and exercises as complementary risk-reduction measures. Planned procedures may not unfold as intended under real emergency conditions, and historical incident data could support comparative, including non-parametric, evaluations of alternative response and evacuation configurations. Third, from a methodological perspective, the bivariate component could be extended to assess co-location between spatial patterns defined across different spatial domains, particularly when one process can occur throughout planar space while another is constrained to a linear network. Such extensions would help connect the present analysis of hydrant infrastructure, incident occurrence, and loss severity to broader questions of fire-risk assessment, preparedness, and the effectiveness of emergency management.

## Supplementary Information

Below is the link to the electronic supplementary material.

Supplementary Material 1

## Data Availability

The majority of the datasets generated and/or analyzed during the current study are available in the Incidents repository, [https://github.com/vanechev29/UrbanFires](https:/github.com/vanechev29/UrbanFires). Incident data are available upon request from the Santa Barbara County Public Records Center: [https://santabarbaracountyca.govqa.us/WEBAPP/_rs/(S(nwoq3s2raog5xdczwf40oycr))/CustomerHome.aspx](https:/santabarbaracountyca.govqa.us/WEBAPP/_rs/(S(nwoq3s2raog5xdczwf40oycr))/CustomerHome.aspx). Hydrant information is available from The Santa Barbara County Fire Department, but restrictions apply to the availability of these data, which were used under license for the current study, and so are not publicly available. Hydrant data are however available from the authors upon reasonable request and with permission of The Santa Barbara County Fire Department.

## References

[1] U.S. Fire Administration. *Residential Fire Estimate Summaries (2014–2023)*. https://www.usfa.fema.gov/statistics/residential-fires/ (2024).

[2] Garrioch, D. 1666 And London’s fire history: A re-evaluation. *Hist. J.* **59**, 319–338 (2016).

[3] Smith, C. *Chicago’s Great Fire: The Destruction and Resurrection of an Iconic American City*. (Atlantic Monthly Press, 2020).

[4] Chhetri, P., Corcoran, J., Ahmad, S. & KC, K. Examining spatio-temporal patterns, drivers and trends of residential fires in South East Queensland, Australia. *Disaster Prev. Management: Int. J.* **27**, 586–603 (2018).

[5] International Code Council. *International Fire Code (IFC 2024)*. https://codes.iccsafe.org/content/IFC2024V2.0 (2024).

[6] National Fire Protection Association. *NFPA 1, Fire Code* (National Fire Protection Association, 2024).

[7] California Fire Code. *International Fire Code (2021 Ed)* (California Fire Code, 2022).

[8] Carpinteria-Summerland Fire Protection District. *Fire Protection Water Supply: Development Standard #2. Carpinteria-Summerland Fire Protection District* (Carpinteria-Summerland Fire Protection District, 2021).

[9] Baker, M. & Browning, K. & Bogel-Burroughs, N. As Inferno Grew, Lahaina’s water system collapsed. *The New. York Times* (2023).

[10] Maranghides, A. et al. WUI Structure/Parcel/Community fire hazard mitigation methodology. (2022).

[11] CAL FIRE. *2025 Incident Archive*. https://www.fire.ca.gov/incidents/2025 (2025).

[12] Moritz, M. A. et al. Learning to coexist with wildfire. *Nature* **515**, 58–66 (2014). 25373675 10.1038/nature13946

[13] Murray, A. T. et al. Coastal vulnerability under extreme weather. *Appl. Spat. Anal. Policy*. **14**, 497–523 (2021).

[14] Syphard, A. D. et al. Human influence on California fire regimes. *Ecol. Appl.* **17**, 1388–1402 (2007). 17708216 10.1890/06-1128.1

[15] Li, S., Dao, V., Kumar, M., Nguyen, P. & Banerjee, T. Mapping the wildland-urban interface in California using remote sensing data. *Sci. Rep.* **12**, 5789 (2022). 35388077 10.1038/s41598-022-09707-7 PMC8987053

[16] Corcoran, J., Higgs, G., Brunsdon, C. & Ware, A. The Use of Comaps to explore the spatial and temporal dynamics of fire incidents: A case study in South Wales, United Kingdom. *Prof. Geogr.* **59**, 521–536 (2007).

[17] Corcoran, J., Higgs, G., Brunsdon, C., Ware, A. & Norman, P. The use of spatial analytical techniques to explore patterns of fire incidence: A South Wales case study. *Comput. Environ. Urban Syst.* **31**, 623–647 (2007).

[18] Jeong, D., Kim, B. & Shin, D. Analysing Spatial Pattern of Fire Factor using Text Mining. *J. Korean Soc. Geospatial Inform. Sci.* **26**, 67–75 (2018).

[19] Rahman Tishi, T. & Islam, I. Urban fire occurrences in the Dhaka Metropolitan Area. *GeoJournal* **84**, 1417–1427 (2019).

[20] Corcoran, J., Higgs, G. & Higginson, A. Fire incidence in metropolitan areas: A comparative study of Brisbane (Australia) and Cardiff (United Kingdom). *Appl. Geogr.* **31**, 65–75 (2011).

[21] Asgary, A., Ghaffari, A. & Levy, J. Spatial and temporal analyses of structural fire incidents and their causes: A case of Toronto, Canada. *Fire Saf. J.* **45**, 44–57 (2010).

[22] Ceyhan, E., Ertuğay, K. & Düzgün, Ş. Exploratory and inferential methods for spatio-temporal analysis of residential fire clustering in urban areas. *Fire Saf. J.* **58**, 226–239 (2013).

[23] Syphard, A. D., Brennan, T. J. & Keeley, J. E. The importance of building construction materials relative to other factors affecting structure survival during wildfire. *Int. J. Disaster Risk Reduct.* **21**, 140–147 (2017).

[24] Alexandre, P. M. et al. The relative impacts of vegetation, topography and spatial arrangement on building loss to wildfires in case studies of California and Colorado. *Landsc. Ecol.* **31**, 415–430 (2016).

[25] Price, O. F., Whittaker, J., Gibbons, P. & Bradstock, R. Comprehensive examination of the determinants of damage to houses in two wildfires in eastern Australia in 2013. *Fire* **4**, 44 (2021).

[26] Maranghides, A. & Mell, W. A case study of a community affected by the witch and Guejito wildland fires. *Fire Technol.* **47**, 379–420 (2011).

[27] Baik, J. & Murray, A. T. Emergency response planning: A framework to assess hydrant-structure access. *Trans. GIS*. **28**, 2412–2424 (2024).

[28] Figueroa, V. E., Murray, A. T. & Funk, T. Supporting Fire Response: Advanced Spatial Data Analytics for Hydrant Access Assessment. *Trans. GIS*. **28**, 2559–2573 (2024).

[29] Singh, P. P., Sabnani, C. S. & Kapse, V. S. Hotspot analysis of structure fires in urban agglomeration: A case of Nagpur City, India. *Fire* **4**, 38 (2021).

[30] Wang, K., Yuan, Y., Chen, M. & Wang, D. A POIs based method for determining spatial distribution of urban fire risk. *Process Saf. Environ. Prot.* **154**, 447–457 (2021).

[31] Bispo, R. et al. Spatial modelling and mapping of urban fire occurrence in Portugal. *Fire Saf. J.* **138**, 103802 (2023).

[32] Gilbert, S. W. & Butry, D. T. Identifying vulnerable populations to death and injuries from residential fires. *Inj. Prev.* **24**, 358–364 (2018). 28774896 10.1136/injuryprev-2017-042343

[33] Shuo, Z., Jingyu, Z., Zhengxiang, Z. & Jianjun, Z. Identifying the density of grassland fire points with kernel density estimation based on spatial distribution characteristics. *Open. Geosci.* **13**, 796–806 (2021).

[34] Guldåker, N. Geovisualization and geographical analysis for fire prevention. *ISPRS Int. J. Geoinf.* **9**, 355 (2020).

[35] Zhang, X., Yao, J., Sila-Nowicka, K. & Jin, Y. Urban fire dynamics and its association with urban growth: Evidence from Nanjing, China. *Int. J. Geo-Information*. **9**, 218 (2020).

[36] Mohammadi, A., Shahparvari, S., Kiani, B., Noori, S. & Chhetri, P. An analysis of Spatio-temporal patterns of fires in an Iranian city. *Indoor Built Environ.* **32**, 183–199 (2023).

[37] Xiong, L., Bruck, D. & Ball, M. Comparative investigation of ‘survival’ and fatality factors in accidental residential fires. *Fire Saf. J.* **73**, 37–47 (2015).

[38] Taylor, M., Francis, H., Fielding, J., Jarman, I. & Etchells, T. A chaos theory view of accidental dwelling fire injuries. *Fire Mater.* **48**, 715–724 (2024).

[39] Xia, Z., Li, H., Chen, Y. & Yu, W. Detecting urban fire high-risk regions using colocation pattern measures. *Sustain. Cities Soc.* **49**, 101607 (2019).

[40] Zhang, Y. Analysis on comprehensive risk assessment for urban fire: The case of Haikou City. *Procedia Eng.* **52**, 618–623 (2013).

[41] Asgary, A., Naini, A. S. & Levy, J. Modeling the risk of structural fire incidents using a self-organizing map. *Fire Saf. J.* **49**, 1–9 (2012).

[42] Corcoran, J., Higgs, G., Rohde, D. & Chhetri, P. Investigating the association between weather conditions, calendar events and socio-economic patterns with trends in fire incidence: an Australian case study. *J. Geogr. Syst.* **13**, 193–226 (2011).

[43] Guldåker, N. & Hallin, P. O. Spatio-temporal patterns of intentional fires, social stress and socio-economic determinants: A case study of Malmö, Sweden. *Fire Saf. J.* **70**, 71–80 (2014).

[44] Kelly, H., Clare, J., Wuschke, K. & Garis, L. Opportunity and rationality as an explanation for suspicious vehicle fires. *Crime. Sci.* **8**, 8 (2019).

[45] KC, K. & Corcoran, J. Modelling residential fire incident response times: A spatial analytic approach. *Appl. Geogr.* **84**, 64–74 (2017).

[46] Todorović, S. K. Modelling risk factors in urban residential fires in Helsinki (Master’s thesis, University of Helsinki, Master’s Program in Geography, Geoinformatics, 2020).

[47] Wang, Z., Zhang, X. & Xu, B. Spatio-Temporal Features of China’s Urban Fires: An investigation with reference to gross domestic product and humidity. *Sustainability* **7**, 9734–9752 (2015).

[48] Hu, J. et al. Socioeconomic determinants of urban fire risk: A city-wide analysis of 283 Chinese cities from 2013 to 2016. *Fire Saf. J.* **110**, 102890 (2019).

[49] Hossain, M. R. & Smirnov, O. Analyzing the risk factors of residential fires in urban and rural census tracts of Ohio using panel data analysis. *Appl. Geogr.* **151**, 102863 (2023).

[50] Rohde, D., Corcoran, J. & Chhetri, P. Spatial forecasting of residential urban fires: A Bayesian approach. *Comput. Environ. Urban Syst.* **34**, 58–69 (2010).

[51] Ardianto, R. & Chhetri, P. Modeling spatial-temporal dynamics of urban residential fire risk using a Markov chain technique. *Int. J. Disaster Risk Sci.* **10**, 57–73 (2019).

[52] Lee, S. L., Hsu, M. H., Wang, Y. F. & Wang, M. Y. Forecasting urban fire severity for enhanced emergency response and resource allocation. *Sci. Rep.* **15**, 42165 (2025). 41298586 10.1038/s41598-025-26006-z PMC12658095

[53] Jain, S. & McLean, C. R. An integrating framework for modeling and simulation for incident management. *J. Homel. Secur. Emerg. Manag.* **3**, (2006).

[54] Himoto, K. & Tanaka, T. Development and validation of a physics-based urban fire spread model. *Fire Saf. J.* **43**, 477–494 (2008).

[55] Halim, S. Z., Quddus, N. & Pasman, H. Time-trend analysis of offshore fire incidents using nonhomogeneous Poisson process through Bayesian inference. *Process Saf. Environ. Prot.* **147**, 421–429 (2021).

[56] Ronchi, E., Gwynne, S. M., Rein, G., Intini, P. & Wadhwani, R. An open multi-physics framework for modelling wildland-urban interface fire evacuations. *Saf. Sci.* **118**, 868–880 (2019).

[57] Silverman, B. W. *Density Estimation for Statistics and Data Analysis* (Chapman and Hall, 1986).

[58] Scott, D. W. *Multivariate Density Estimation: Theory, Practice, and Visualization* (Wiley, 1992).

[59] Diggle, P. J. *Statistical Analysis of Spatial and Spatio-Temporal Point Patterns (3rd Ed.)*. (Chapman & Hall/CRC, 2013).

[60] Diggle, P. J. A kernel method for smoothing point process data. *J. R Stat. Soc. Ser. C Appl. Stat.* **34**, 138–147 (1985).

[61] Sheather, S. J. & Jones, M. C. A Reliable Data-Based Bandwidth Selection Method for Kernel Density Estimation. *J. R Stat. Soc. Ser. B Stat. Methodol.* **53**, 683–690 (1991).

[62] Ripley, B. D. The second-order analysis of stationary point processes. *J. Appl. Probab.* **13**, 255–266 (1976).

[63] Ripley, B. D. Modelling spatial patterns. *J. Royal Stat. Soc. Ser. B (Methodological)*. **39**, 172–192 (1977).

[64] Besag, J. Contribution to the discussion on Dr Ripley’s paper. *J. Royal Stat. Soc. Ser. B (Methodological)*. **39**, 193–195 (1977).

[65] Ripley, B. D. Tests of ‘randomness’ for spatial point patterns. *J. Royal Stat. Soc. Ser. B (Methodological)*. **41**, 368–374 (1979).

[66] Raczek, J. & Miotk, M. Theoretical modelling of efficient fire safety water networks by certified domination. *Sci. Rep.* **14**, 21574 (2024). 39284835 10.1038/s41598-024-72285-3 PMC11405400

