# Supplementary material for: Urban safety and the role of hydrant structure and configuration
Source: Sci Rep. 2026 Apr 11;16:17049. doi: 10.1038/s41598-026-45799-1 (PMC13230598; doi:10.1038/s41598-026-45799-1)
Supplement: Supplementary file 1 — Supplementary Material 1 [file 41598_2026_45799_MOESM1_ESM.docx]

**Table 3.** **Gamma regressions with a log link.**

| $Y=$ Loss (USD); Gamma(log); $n=90$ | **Model 1** | **Model 2** | **Model 3** | **Model 4** | **Model 5** |
| --- | --- | --- | --- | --- | --- |
| (Intercept) | 2.956  (3.151) | 4.770  (3.354) | 4.098 (3.215) | 4.537 (3.174) | 4.724 (3.385) |
| *lh* | -0.038  (0.257) | -0.243  (0.273) | -0.393 (0.259) | -0.435 (0.250) | -0.495 (0.258) |
| *la* | 0.542**  (0.172) | 0.408** (0.182) | 0.310 (0.174) | 0.260 (0.170) | 0.215 (0.177) |
| *lf* | -0.076  (0.350) | -0.024  (0.357) | 0.202 (0.320) | 0.176 (0.301) | 0.233 (0.314) |
| *lt* | 1.213***  (0.185) | 1.228***  (0.188) | 1.220*** (0.181) | 1.330*** (0.187) | 1.326*** (0.189) |
| *Hyd-to-str*  (baseline: noncompliant) | 0.001  (0.411) | — | — | — | — |
| **Type of building (baseline: residential)** | | | | | |
| Commercial | — | 0.260  (0.636) | 0.350 (0.581) | 0.344 (0.546) | 0.223 (0.550) |
| Institutional | — | 1.540  (1.674) | 1.529 (1.584) | 1.576 (1.510) | 1.990 (1.545) |
| Outbuilding | — | -1.317*  (0.669) | -1.431* (0.633) | -1.739** (0.643) | -1.799** (0.686) |
| **Time of the day (baseline afternoon)** | | | | | |
| Dawn | — | — | 1.454* (0.607) | 1.363* (0.616) | 1.463* (0.651) |
| Morning | — | — | 0.200 (0.554) | 0.303 (0.577) | 0.386 (0.579) |
| Lunch Time | — | — | 0.100 (0.726) | -0.063 (0.738) | -0.061 (0.767) |
| Evening | — | — | -0.952 (0.625) | -1.116 (0.667) | -0.941 (0.689) |
| Night | — | — | 0.393 (0.554) | 0.426 (0.591) | 0.424 (0.596) |
| **Day of the week (baseline Friday)** | | | | | |
| Monday | — | — | — | -0.134 (0.792) | -0.101 (0.829) |
| Tuesday | — | — | — | -0.153 (0.691) | 0.025 (0.688) |
| Wednesday | — | — | — | 0.023 (0.657) | 0.193 (0.656) |
| Thursday | — | — | — | 0.510 (0.670) | 0.693 (0.676) |
| Saturday | — | — | — | 1.304 (0.762) | 1.634* (0.802) |
| Sunday | — | — | — | -0.337 (0.734) | -0.186 (0.722) |
| **Season of the year (baseline Winter)** | | | | | |
| Spring | — | — | — | — | -0.373 (0.502) |
| Summer | — | — | — | — | 0.189 (0.543) |
| Fall | — | — | — | — | -0.421 (0.441) |
| AIC | 2084.9 | 2084.29 | 2080.51 | 2083.94 | 2088.17 |
| BIC | 2104.9 | 2109.29 | 2118.01 | 2136.43 | 2148.17 |
| logLik | -1034.4 | -1032.15 | -1025.25 | -1020.97 | -1020.09 |
| $R^{2}$ (marginal) | 0.432 | 0.457 | 0.571 | 0.638 | 0.648 |
| $R^{2}$ (conditional) | 0.774 | 0.768 | 0.796 | 0.781 | 0.787 |
| ICC (adjusted) | 0.602 | 0.573 | 0.524 | 0.397 | 0.395 |
| DHARMa KS (D, p) | 0.11, 0.26 | 0.07, 0.72 | 0.08, 0.61 | 0.06, 0.88 | 0.07, 0.80 |
| DHARMa Disp (stat, p) | 1.16, 0.27 | 3.36, 0.14 | 0.79, 0.30 | 1.41, 0.27 | 2.24, 0.17 |
| DHARMa Out (stat, p) | 1.00, 0.51 | 0.00, 1.00 | 1.00, 0.51 | 0.00, 1.00 | 1.00, 0.51 |
| DHARMa Quant (p) | 0.212 | 0.138 | 0.316 | 0.310 | 0.074 |
| Standard errors in parentheses. Significance *p < 0.05 *, p < 0.01 **, p < 0.001 ***.* *lh:* distance to the nearest hydrant; *la:* structure area *lf:* distance to the nearest fire station; *lt:* total suppression time; *Hyd-to-str:* hydrant to structure access. | | | | | |
